# Influence of Scleral Contact Lenses on Optical Coherence Tomography Parameters in Keratoconus Patients

**DOI:** 10.3390/diagnostics15192541

**Published:** 2025-10-09

**Authors:** Atılım Armağan Demirtaş, Aytül Arslan, Berna Yüce, Tuncay Küsbeci

**Affiliations:** 1 Department of Ophthalmology, Izmir Faculty of Medicine, University of Health Sciences Turkey, Izmir 35540, Turkey; brnyuce@yahoo.com (B.Y.); tkusbeci@yahoo.com (T.K.); 2 Department of Ophthalmology, Izmir City Hospital, Izmir 35540, Turkey; aytularslan405@gmail.com

**Keywords:** corneal thickness, ganglion cell-inner plexiform layer, keratoconus, optical coherence tomography, quality index, retinal nerve fiber layer, scleral contact lenses

## Abstract

**Background**: This study aimed to evaluate the influence of scleral contact lens (SCL) wear on optical coherence tomography (OCT) scan quality and structural measurements in patients with keratoconus. **Methods**: This retrospective observational study included 28 eyes of 28 keratoconus patients. All participants underwent a comprehensive ophthalmologic evaluation, including corneal topography and spectral-domain OCT (Optopol REVO 60). Two OCT measurement sessions were performed on the same day: one without SCLs and one after a 30–75 min adaptation period with Mini Misa^®^ scleral lenses. Recorded parameters included corneal and epithelial thicknesses, ganglion cell–inner plexiform layer (GCIPL) thickness, retinal nerve fiber layer (RNFL) thickness, and device-reported quality index (QI). Correlation analyses between topographic values, age, and OCT parameters were also conducted. **Results**: The mean age of participants was 32.96 ± 13.72 years. SCL wear significantly decreased anterior segment QI (6.76 ± 1.73 vs. 5.57 ± 2.34, *p* = 0.019) but improved posterior segment QI in both the ganglion (2.52 ± 1.03 vs. 5.76 ± 2.17, *p* < 0.001) and disc (2.82 ± 0.94 vs. 4.39 ± 1.87, *p* < 0.001) modules. Central corneal thickness remained stable, while central epithelial thickness decreased slightly (50.53 ± 6.66 µm vs. 47.59 ± 7.20 µm, *p* = 0.007). RNFL and GCIPL thicknesses showed no significant changes, except for minor sectoral variations. Steeper keratometry values correlated with lower QI in both conditions. **Conclusions**: SCLs enhanced posterior OCT scan quality while reducing anterior segment image clarity. These findings suggest that SCLs not only provide visual rehabilitation but also facilitate more reliable posterior segment imaging in keratoconus patients, despite mild interference with anterior segment OCT metrics. Further prospective studies are warranted to validate these results.

## 1. Introduction

Keratoconus is a progressive, bilateral corneal ectatic disorder characterized by localized thinning and conical protrusion, leading to high myopia and notably irregular astigmatism that cannot be fully corrected with conventional lenses, resulting in significantly impaired visual acuity and quality of life [1,2,3,4,5]. Corneal topography remains one of the most frequently used tools for the diagnosis and follow-up of keratoconus, and Pentacam^®^ parameters have been evaluated under different grading definitions to improve the detection of subclinical and mild cases [6].

In keratoconus management, rigid contact lenses—including scleral contact lenses (SCLs)—are crucial for visual rehabilitation. Unlike corneal lenses, SCLs vault over the cornea and rest on the sclera, creating a tear-filled reservoir that neutralizes corneal irregularities, improves visual acuity, and enhances comfort for patients with advanced or intolerant corneal profiles [7,8,9].

Optical coherence tomography (OCT) is a non-invasive imaging tool offering micrometer-level resolution by using low-coherence interferometry, making it invaluable for assessing anterior and posterior segment structures rapidly and safely [10,11]. However, in keratoconus, the presence of irregular astigmatism may compromise OCT image quality and segmentation accuracy, thereby complicating the acquisition of reliable data in real-world conditions [5,12,13,14,15].

Given that SCLs improve optical regularity, they may also enhance OCT image quality and measurement accuracy for both anterior and posterior segments by stabilizing the tear reservoir and smoothing the refractive surface. Some emerging evidence supports this potential, though studies specifically exploring OCT outcomes with SCL wear remain limited.

Therefore, the present study aims to investigate how SCLs affect OCT scan quality and structural measurements—particularly corneal thickness, epithelial thickness, ganglion cell-inner plexiform layer (GCIPL) thickness, and retinal nerve fiber layer (RNFL) thickness—in keratoconus patients.

## 2. Materials and Methods

### 2.1. Study Design and Ethics

This study was designed as a retrospective, observational study and was conducted at the Izmir City Hospital. A total of 28 patients diagnosed with keratoconus were included. Only one eye from each patient was selected, resulting in a total of 28 eyes analyzed. The study protocol was reviewed and approved by the Non-Interventional Clinical Research Ethics Committee of Izmir City Hospital (decision date: 23 July 2025, no: 2025/362), and written informed consent was obtained from all participants before enrollment. All procedures were carried out in accordance with the Declaration of Helsinki, ensuring that the ethical principles of medical research were strictly followed.

### 2.2. Patients

Participants were required to be 18 years of age or older and to have a confirmed diagnosis of keratoconus based on clinical findings and corneal topography. Only eyes with a clear central cornea and no history of ocular surgery, including corneal collagen cross-linking, were eligible for inclusion.

Exclusion criteria included corneal scarring or opacities, systemic or ocular surface diseases affecting the cornea. Both eyes of each participant were evaluated during the initial examination, but to avoid inter-eye correlation, only one randomly selected eye per participant was included in the final analysis. Additionally, individuals with a history of either soft or rigid contact lens wear were excluded to eliminate potential confounding effects on corneal measurements.

All participants underwent a comprehensive ophthalmologic examination. This included the measurement of uncorrected visual acuity (UCVA) and best-corrected visual acuity (BCVA) measured at a distance of 6 m using a standard Snellen chart, slit-lamp biomicroscopy, fundus examination, intraocular pressure measurement using Goldmann applanation tonometry, and corneal tomography imaging using the Pentacam^®^ Scheimpflug camera (Oculus, Wetzlar, Germany) to confirm keratoconus diagnosis.

### 2.3. Scleral Contact Lens Details and Fitting Process

The study utilized Mini Misa SCLs (Mini Scleral^®^, Microlens, Arnhem, The Netherlands) are designed to offer precise corneal vaulting and stable scleral support, with several customizable parameters to suit individual patient anatomy. The lenses are available in different total diameters (16.5, 17.0, and 17.5 mm) and feature a base curve radius that can be finely adjusted to achieve an optimal fit. Vault height can be tailored in small increments to ensure appropriate central corneal clearance. The optical zone is standardized at 8.0 mm to provide consistent visual quality, while power options range from high minus to high plus values, with toric designs available for astigmatic correction. The lenses have a central thickness of approximately 300 µm, balancing structural stability with adequate oxygen permeability.

Mini Misa trial lenses were used during the initial fitting process, and all fittings were performed by an experienced ophthalmologist specialized in contact lens fitting (A.A.D.). The fitting process began by selecting the most appropriate vault height, which was determined using the trial lens set provided by the Misa^®^ system to ensure adequate corneal clearance. After lens insertion, the fit was evaluated exclusively with slit-lamp biomicroscopy using fluorescein dye to assess the distribution of the tear film beneath the lens and to detect any areas of excessive bearing or excessive clearance. The target central vault was approximately 200 µm after the lens had settled. Lens centration, limbal clearance, and peripheral alignment were carefully examined to achieve a stable and uniform fit. Once the optimal lens fit was established, over-refraction was performed through the diagnostic lens to refine the final lens power for precise visual correction. Final lens parameters, including base curve radius, vault height, total diameter, and power, were customized for each participant and recorded.

### 2.4. Optical Coherence Tomography Assessments

All imaging procedures were performed using the Optopol REVO 60 Spectral Domain (SD) OCT device (Optopol Technology, Zawiercie, Poland; Software Version 11.5.0). This system provides high-resolution images with an axial resolution of 5 µm and operates at a scanning speed of 60,000 A-scans per second, allowing for detailed visualization of both anterior and posterior segment structures. For all examinations, no additional lens was required.

### 2.5. Examination Protocol

For each participant, two sets of OCT scans were obtained on the same day. Imaging sessions were carried out without pharmacological pupil dilation. The first set of OCT measurements was taken without contact lenses, following a brief period of rest to ensure tear film stability. After the initial measurements, SCLs were inserted and evaluated to confirm adequate fitting. Participants then underwent a 30–75 min adaptation period with the lenses in place. Following this adaptation period, the second set of OCT scans was performed while the lenses were being worn. Before each OCT acquisition, patients were instructed to blink naturally to ensure a smooth optical surface and consistent image quality. To ensure consistency, both lens-on and lens-off OCT measurements were performed under appropriate ambient lighting and climate-controlled room conditions, in line with best practices in OCT imaging [16], and care was taken to capture well-centered, high-quality images in both conditions. The scan QI provided by the device was recorded for each module in both conditions to assess whether SCL wear influenced image quality. The QI index is an integrated parameter provided by the OCT software (version 11.5.0), reflecting the signal strength and image reliability based on factors such as signal-to-noise ratio and scan centration. According to the manufacturer’s guidelines and previous reports, scans with a QI ≥ 2 and without segmentation errors or other evident artifacts were considered to be of acceptable quality and were included in the analysis. All imaging procedures were carried out by the same experienced technician, following a standardized order: anterior segment imaging, followed by ganglion and disc measurements. For both lens-on and lens-off conditions, measurements in each module were repeated twice, and the scans with the higher QI and no imaging artifacts were selected for statistical analysis.

### 2.6. Parameters

Baseline characteristics recorded for each participant included age and gender. For each eye, the following data were collected: laterality (right or left), UCVA, and BCVA. Corneal topographic parameters were documented, including flat keratometry (K1), steep keratometry (K2), mean keratometry (Km), maximum keratometry (Kmax), and corneal astigmatism.

Anterior segment OCT measurements included central, maximum, and minimum corneal thickness, as well as central, maximum, and minimum epithelial thickness. Ganglion analysis included average and minimum GCIPL thickness, along with six sectoral measurements: superotemporal, superior, superonasal, inferonasal, inferior, and inferotemporal. Disc analysis included disc area, vertical cup to disc (C/D) ratio, average RNFL thickness, and quadrant-specific RNFL values (superior, inferior, nasal, and temporal). In all OCT modules, the QI was recorded for statistical evaluation.

### 2.7. Data Analyses

A power analysis indicated that a sample size of 28 eyes was required to detect a medium effect size (Cohen’s d = 0.76) with a statistical power of 80% and a significance level of α = 0.05 (E-PICOSAI, MedicRes, New York, NY, USA). Data were analyzed using IBM SPSS Statistics Version 21.0 (IBM Corp., Armonk, NY, USA). Paired measurements between lens-off and lens-on conditions were compared using the paired *t*-test for normally distributed variables and the Wilcoxon signed-rank test for non-normally distributed variables. Correlation analyses were performed using Spearman’s test. A two-sided *p*-value < 0.05 was considered statistically significant.

## 3. Results

A total of 28 eyes from 28 patients diagnosed with keratoconus were evaluated, including 18 males (64.3%) and 10 females (35.7%). The mean age of the participants was 32.96 ± 13.72 years (median: 27 years; range: 18–60 years). Among the examined eyes, 17 (60.7%) were right eyes and 11 (39.3%) were left eyes. The mean UCVA was 0.24 ± 0.23, while the mean BCVA showed a significant improvement to 0.86 ± 0.24 (*p* < 0.001).

### 3.1. Corneal Topographic Values

The mean K1 was 47.71 ± 6.08 diopter (D), and the K2 was 51.83 ± 6.74 D, with a mean Km of 49.77 ± 4.54 D. The mean Kmax was 59.16 ± 9.06 D, and the mean corneal astigmatism was 4.01 ± 2.30 D (range: 1.1–10.3 D).

### 3.2. Changes in OCT Measurements With and Without Scleral Contact Lenses

**Figure 1:**
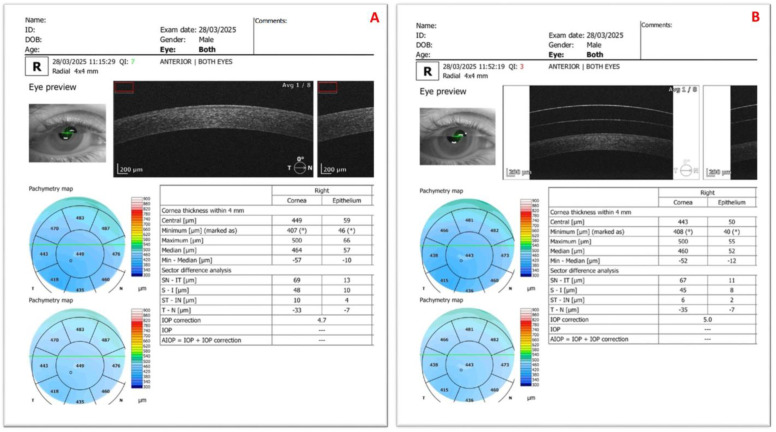
Anterior segment optical coherence tomography images of the right eye of a 27-year-old male patient with keratoconus, showing (**A**) without scleral contact lens wear and (**B**) after scleral contact lens application. The patient’s visual acuity improved from 0.6 (uncorrected) to 1.0 (corrected) with the scleral contact lens. The symbol (°) on the corneal thickness (pachymetry) map indicates the thinnest point of the cornea, whereas on the epithelial thickness map (not included) the thinnest location is marked with an asterisk (*).

**Figure 2:**
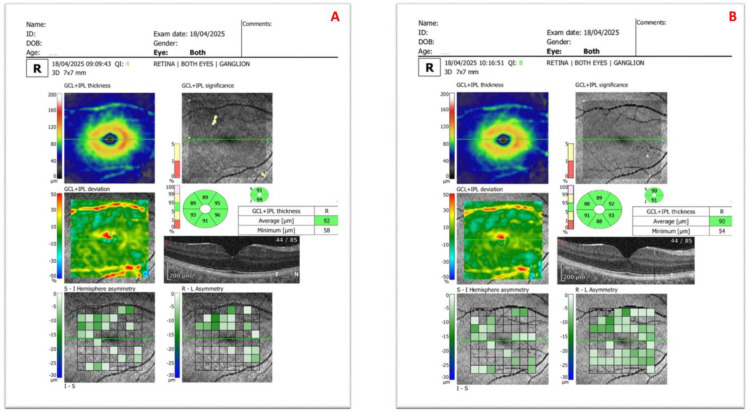
Ganglion optical coherence tomography images of the right eye of a 22-year-old male patient with keratoconus, showing (**A**) without scleral contact lens wear and (**B**) after scleral contact lens application. The patient’s visual acuity improved from 0.2 (uncorrected) to 1.0 (corrected) with the scleral contact lens.

**Figure 3:**
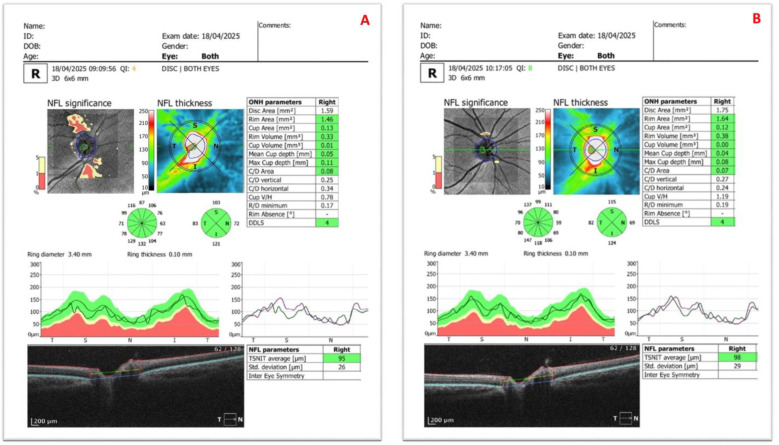
Disc optical coherence tomography images of the right eye of a 24-year-old female patient with keratoconus, showing (**A**) without scleral contact lens wear and (**B**) after scleral contact lens application. The patient’s visual acuity improved from 0.1 (uncorrected) to 1.0 (corrected) with the scleral contact lens.

Figure 1, Figure 2 and Figure 3 present representative anterior segment, ganglion, and disc OCT scans from participants, each acquired without and with SCLs.

Changes in OCT measurements without SCLs and with SCLs are presented in Table 1.

In the anterior segment module, the mean QI significantly decreased when SCLs were worn (6.76 ± 1.73 without lenses vs. 5.57 ± 2.34 with lenses; *p* = 0.019). Central corneal thickness remained stable between conditions (431.05 ± 50.73 µm vs. 431.05 ± 55.28 µm; *p* = 0.794). No significant differences were observed for maximum or minimum corneal thickness values (*p* > 0.05). In contrast, central epithelial thickness decreased significantly with SCL wear (50.53 ± 6.66 µm vs. 47.59 ± 7.20 µm; *p* = 0.007), as did minimum epithelial thickness values (*p* = 0.002 for both).

For the ganglion analysis, QI was markedly higher with lenses compared to without (5.76 ± 2.17 vs. 2.52 ± 1.03; *p* < 0.001). Average and minimum GCIPL thicknesses did not differ significantly between the two conditions (*p* > 0.05). Among the sectoral GCIPL measurements, only the inferior sector showed a significant reduction with SCL wear (87.32 ± 5.78 µm vs. 86.05 ± 5.72 µm; *p* = 0.001).

In the disc module, QI improved significantly with lenses (4.39 ± 1.87 vs. 2.82 ± 0.94; *p* < 0.001). The average RNFL thickness remained comparable between conditions (93.15 ± 12.66 µm vs. 93.74 ± 9.51 µm; *p* = 0.070), though the superior quadrant showed a significant increase with SCL wear (106.50 ± 18.33 µm vs. 112.71 ± 16.07 µm; *p* = 0.005). Temporal RNFL thickness decreased slightly but significantly (*p* = 0.024), while nasal and inferior quadrants showed no meaningful changes. Disc area and vertical C/D ratio were similar between conditions (*p* > 0.05).

### 3.3. Correlation Analyses

Correlation analyses between age, topographic values, and OCT measurements without SCLs and with SCLs are presented in Table 2 and Table 3, respectively.

In eyes without SCLs, anterior segment analysis showed that higher K1 and Kmax values were associated with lower image QI values (K1: r = −0.393, *p* = 0.039; Kmax: r = −0.471, *p* = 0.011). Kmax was positively correlated with maximum (r = 0.395, *p* = 0.038) corneal thickness, whereas astigmatism was positively associated with central (r = 0.394, *p* = 0.038) and minimum (r = 0.393, *p* = 0.038) corneal thickness. Regarding epithelial measurements, central epithelial thickness was significantly lower with increasing K1 (r = −0.501, *p* = 0.007) and K2 (r = −0.483, *p* = 0.009), while maximum epithelial thickness decreased with higher K2 values (r = −0.388, *p* = 0.041). In the ganglion analysis, QI decreased with increasing K2 (r = −0.460, *p* = 0.021) and Kmax (r = −0.454, *p* = 0.023). Additionally, age was negatively correlated with GCIPL thickness in the superior (r = −0.449, *p* = 0.031), superior temporal (r = −0.432, *p* = 0.039), inferior temporal (r = −0.436, *p* = 0.037), and minimum (r = −0.425, *p* = 0.043) sectors. In the disc analysis, average RNFL thickness showed significant negative correlations with K1 (r = −0.552, *p* = 0.003) and Kmax (r = −0.498, *p* = 0.008). Nasal RNFL thickness also decreased with increasing K1 (r = −0.509, *p* = 0.006) and Kmax (r = −0.425, *p* = 0.024). Inferior (r = −0.476, *p* = 0.010) and temporal (r = −0.494, *p* = 0.014) RNFL thickness showed similar negative correlations with Kmax. Disc QI was negatively associated with K2 (r = −0.384, *p* = 0.044) and Kmax (r = −0.403, *p* = 0.033). A positive correlation was found between astigmatism and nasal RNFL thickness (r = 0.444, *p* = 0.018). Disc area was positively correlated with astigmatism (r = 0.463, *p* = 0.013).

In eyes with SCLs, anterior segment analysis showed QI demonstrated a negative correlation with K1 (r = −0.428, *p* = 0.021), K2 (r = −0.412, *p* = 0.025), and Kmax (r = −0.437, *p* = 0.019), indicating reduced image quality with steeper corneal curvatures. Minimum corneal thickness was positively correlated with Kmax (r = 0.396, *p* = 0.031) and astigmatism (r = 0.381, *p* = 0.038). Increasing age was associated with greater central epithelial thickness (r = 0.418, *p* = 0.024), and maximum epithelial thickness (r = 0.442, *p* = 0.018). Higher K1 (r = −0.409, *p* = 0.026) and K2 (r = −0.397, *p* = 0.030) values were linked to thinner central corneal thickness, while K2 was positively correlated with maximum corneal thickness (r = 0.384, *p* = 0.036). In ganglion analysis, no significant correlations were observed between age, topographic parameters, and OCT measurements (all *p* > 0.05). For the disc analysis, QI showed a negative correlation with age (r = −0.421, *p* = 0.023) and a positive correlation with astigmatism (r = 0.412, *p* = 0.026). Average RNFL thickness decreased significantly as K1 (r = −0.453, *p* = 0.015), K2 (r = −0.438, *p* = 0.018), and Kmax (r = −0.469, *p* = 0.013) increased. Superior RNFL thickness declined with advancing age (r = −0.402, *p* = 0.028). Nasal RNFL thickness showed a negative correlation with K1 (r = −0.391, *p* = 0.032) and Kmax (r = −0.417, *p* = 0.024), while a positive correlation was found with astigmatism (r = 0.404, *p* = 0.027). Disc area was negatively correlated with K1 (r = −0.444, *p* = 0.018).

## 4. Discussion

This study evaluated the effect of SCL wear on anterior and posterior segment OCT parameters in patients with keratoconus. Our results demonstrated that while SCLs did not significantly alter corneal thickness, they caused a notable decrease in the anterior segment scan QI and a significant increase in the posterior segment scan QI, particularly in the ganglion and disc modules. Additionally, central epithelial thickness decreased significantly with SCL wear, whereas GCIPL and RNFL thickness values remained largely stable except for subtle sectoral changes.

Anterior segment OCT is a key non-invasive tool for diagnosing and monitoring keratoconus, as it enables detailed mapping of pachymetry and epithelial thickness [17,18]. Subtle epithelial patterns can be detected even before topographic abnormalities emerge [19], and recent advances such as epithelial backscatter analysis and swept-source OCT have further strengthened its diagnostic and follow-up utility [20,21,22].

In our study, lens-off OCT scans demonstrated that steeper corneas and higher astigmatism were associated with reduced image quality and characteristic epithelial thinning patterns. These findings support the notion that keratoconus severity influences both imaging reliability and corneal morphology.

Previous studies have reported posterior segment changes in keratoconus, including RNFL thinning, localized ganglion cell complex alterations, and mild macular changes, suggesting that irregular astigmatism can influence retinal and optic nerve measurements [14,23,24,25]. Our results align with these findings, showing significant associations between corneal topographic severity indices and OCT-derived posterior parameters. Without scleral lenses, in posterior segment modules, higher keratometric values were associated with lower QI, thinner RNFL measurements, and sectoral GCIPL thinning, indicating that disease severity affects both structural integrity and imaging reliability.

When SCLs were worn, anterior segment QI continued to decline with steeper corneal curvatures, similar to the lens-off condition, suggesting that even with optical neutralization, highly irregular corneas remain challenging for image acquisition. Minimum corneal thickness correlated positively with Kmax and astigmatism, while thinner central corneal thickness was linked to steeper K values, consistent with the natural thinning pattern of keratoconus. Unlike the lens-off state, no significant correlations were observed between ganglion parameters and topographic values, suggesting that SCLs help stabilize macular measurements by improving posterior segment image quality. However, in the disc analysis, average and superior RNFL thickness still declined with increasing K values, indicating that optic nerve measurements remain sensitive to underlying corneal biomechanics. These findings highlight that while SCLs improve scan quality and reduce some artifacts, they do not fully eliminate the influence of corneal irregularity on posterior segment OCT metrics.

The current literature has primarily focused on the effects of soft contact lenses on OCT image quality and measurement accuracy, mainly in healthy individuals or those with myopia. Findings from these studies are inconsistent, with some reporting a slight reduction or no effect on scan quality with soft lens wear, while others suggest that soft lenses may improve measurement reliability in highly myopic eyes by creating a smoother refractive surface [26,27,28,29,30]. In contrast, there is limited data on rigid contact lenses, particularly SCLs, in keratoconus patients. Only a few studies, such as the work by Uzunel et al., have explored OCT measurements with and without rigid gas-permeable corneal lenses. In their study, 52 eyes from 31 keratoconus patients were evaluated using Cirrus HD-OCT, and significant increases were observed in average RNFL thickness, nasal quadrant thickness, several clock-hour sectors, and central macular thickness, along with improved signal strength. These results highlight the importance of addressing irregular astigmatism to achieve reliable OCT measurements in keratoconic eyes [31].

Our study most closely parallels the work of Pinheiro-Costa et al., who examined whether the irregular astigmatism and optical aberrations associated with keratoconus influence the accuracy of macular region measurements using Spectralis OCT. In their study, 24 keratoconic eyes were evaluated both without SCLs after a 48 h cessation period and again 15 min after lens application. They found no statistically significant differences in macular thickness or volume between the lens-on and lens-off conditions, with *p*-values ranging from 0.114 to 0.944. Although there was a slight, non-significant decrease in central subfield thickness with lens wear (*p* = 0.111), the overall results indicated that SCLs did not significantly alter fovea-centered macular measurements. These findings suggest that, unlike RNFL analysis, macular measurements are relatively resistant to the optical irregularities caused by keratoconus [32]. Similarly, in our study, GCIPL thickness values remained largely stable between lens-off and lens-on conditions, with only minor sectoral variations observed. This parallels the findings of Pinheiro-Costa et al., suggesting that while SCLs substantially improve posterior scan quality by neutralizing corneal irregularities, they do not induce significant short-term structural changes in the macular or ganglion cell regions. However, our study extends these results by demonstrating that the improvement in scan quality provided by SCLs is most pronounced in the posterior segment modules, where reliable imaging is often difficult to achieve in keratoconus due to severe corneal irregularity.

The impact of astigmatism on OCT-based RNFL measurements has been extensively investigated, with studies consistently indicating that certain optic nerve quadrants are more influenced depending on both the magnitude and axis of astigmatism [27,33,34]. Previous research has demonstrated that while macular thickness measurements remain largely unaffected by refractive factors such as myopia and astigmatism, RNFL analysis can be significantly influenced by these conditions [33,34]. In particular, Hwang et al. found that inducing astigmatism through toric soft contact lenses in healthy individuals led to notable changes in RNFL thickness, whereas macular thickness measurements remained stable, suggesting that astigmatism primarily impacts peripapillary rather than macular OCT evaluations [34]. However, in our study, while GCIPL thickness remained unchanged as noted earlier, RNFL thickness values also showed no significant differences between lens-off and lens-on conditions, with only minor sectoral variations observed.

To date, no published studies have comprehensively assessed the impact of SCLs on both anterior and posterior segment OCT parameters in keratoconus. This represents a notable gap, as the irregular astigmatism characteristic of keratoconus often reduces scan quality and introduces segmentation errors. In our study, SCL wear markedly improved posterior segment scan quality and stabilized RNFL and GCIPL measurements, indicating that the tear-filled reservoir created by the lens effectively neutralizes corneal irregularities. Conversely, a slight decrease in anterior segment QI was observed, likely due to optical scattering caused by the lens material, tear reservoir, and backscatter effects. This reduction may also be partially attributed to early fogging phenomena; however, given that the lenses were worn for only 30–75 min, the development of midday fogging [35,36,37], which typically occurs after several hours, was unlikely.

Additionally, our results showed that corneal thickness parameters, including central corneal thickness, remained stable between lens-off and lens-on conditions, suggesting that the SCL vaulting mechanism does not exert mechanical pressure sufficient to alter corneal structure in the short term. In contrast, epithelial thickness demonstrated a small but statistically significant decrease after lens wear, which may represent early redistribution in response to lens-induced changes in tear film dynamics, oxygen diffusion, or mechanical stress. The corneal epithelium is known to rapidly remodel—thinning over elevated regions and thickening in adjacent depressed zones—as part of its smoothing function over stromal irregularities; such behavior has been documented in keratoconus and post-refractive surgery contexts [38,39]. In contact lens wear studies, epithelial thickness modulation over time has been observed, likely reflecting adaptation to altered biomechanical or metabolic conditions [40]. Future studies with serial imaging over longer wear periods might capture the kinetics of these epithelial adjustments in keratoconic eyes wearing scleral lenses. Since minimum corneal thickness is a critical marker for monitoring keratoconus progression, the stability of these measurements reinforces the reliability of longitudinal OCT follow-up, even in the presence of SCL wear.

In OCT-based studies, scan quality is a critical determinant of measurement accuracy. Different devices employ varying indices and thresholds [41,42,43,44]. For example, Cirrus HD-OCT (Zeiss) uses a Signal Strength (SS) scale of 0–10, with values ≥ 6 recommended for clinical use, while Spectralis OCT (Heidelberg Engineering) employs a Q-score (0–40) with ≥20 considered acceptable. RTVue OCT (Optovue) uses a Signal Strength Index (SSI), where values above 40–45 indicate high-quality scans. The Optopol REVO OCT, used in our study, employs a QI scale of 0–10, with ≥4 recommended for reliable measurements [44]. By setting a threshold of QI ≥ 2, we aimed to capture clinically usable scans while reflecting the real-world difficulties encountered in keratoconus imaging.

OCT in keratoconus should not be performed routinely with SCLs, but lens-on imaging can be valuable in selected situations. Lens-off anterior segment OCT remains the standard for diagnosis, staging, and follow-up, as lens wear may transiently alter epithelial measurements. However, lens-on OCT may improve scan quality in advanced keratoconus with severe irregularity, poor fixation, or segmentation errors, particularly for posterior segment evaluation. Moreover, despite the minor decrease in anterior segment scan quality, lens-on OCT can still provide useful information for lens fitting, including vault height and scleral landing zone positioning, thereby complementing the initial slit-lamp biomicroscopy evaluation [45,46,47].

This study has several limitations. First, its retrospective design limits causal inference, and the relatively small sample size may reduce statistical power. While regression models could have provided additional insights into the interaction between the studied parameters, due to this limited sample size and the scope of the current dataset, performing a meaningful regression analysis was not feasible. Second, while most included eyes were stage 2 (mean Km = 49.77 ± 4.54 D) keratoconus according to the Amsler Krumeich grading system, the sample remained heterogeneous, and a subgroup analysis comparing early versus advanced disease was not feasible; this may have influenced the variability of OCT measurements. Additionally, SCL wear was evaluated only during short-term adaptation (30–75 min); longer-term effects, including midday fogging and epithelial remodeling, were not assessed. Furthermore, only a single OCT device (Optopol REVO) was used, which may limit the generalizability of our findings to other imaging systems. The absence of a healthy control group and lack of long-term follow-up data are also important limitations. Finally, no prior studies have evaluated OCT scan quality and anterior & posterior structural parameters in keratoconus patients wearing SCLs, making direct comparisons difficult. Future prospective studies with larger cohorts, healthy control groups, multiple imaging platforms, and longitudinal follow-up are needed to validate and expand upon these findings.

## 5. Conclusions

Our results suggest that SCL wear improves posterior segment OCT scan quality and measurement reliability in keratoconus patients while slightly reducing anterior segment QI. These lenses provide both visual rehabilitation and enhanced imaging conditions for patients with irregular astigmatism. Given the increasing use of OCT in keratoconus monitoring and treatment planning, incorporating SCL wear during imaging could improve diagnostic accuracy and patient care. This study represents an important initial step in understanding the dual functional and diagnostic benefits of scleral contact lenses.

## Figures and Tables

**Table 1:** Changes in OCT measurements with and without scleral contact lenses.

	Mean ± SD			*p*
	Without Lenses (*n* = 28)	With Lenses (*n* = 28)	Difference (*n* = 28)	
**Anterior segment analysis**				
QI	6.76 ± 1.73	5.57 ± 2.34	−1.19 ± 2.14	**0.019 ^1^**
Corneal thickness (µm)				
Central	431.05 ± 50.73	431.05 ± 55.28	0.0 ± 9.52	0.794 ^2^
Maximum	512.29 ± 41.08	510.35 ± 41.14	−1.94 ± 20.01	0.694 ^1^
Minimum	392.81 ± 56.34	382.95 ± 73.49	−9.86 ± 30.76	0.643 ^2^
Epithelial thickness (µm)				
Central	50.53 ± 6.66	47.59 ± 7.2	−2.94 ± 3.65	**0.007 ^2^**
Maximum	63.24 ± 10.87	62.24 ± 18.18	−1.0 ± 10.71	0.093 ^2^
Minimum	41.65 ± 4.82	38.29 ± 4.67	−3.35 ± 3.69	**0.002 ^1^**
**Ganglion analysis**				
QI	2.52 ± 1.03	5.76 ± 2.17	3.24 ± 2.05	**<0.001 ^1^**
GCIPL thickness (µm)				
Average	86.59 ± 5.55	86.09 ± 4.7	−0.5 ± 1.87	0.153 ^2^
Minimum	42.55 ± 8.86	44.64 ± 8.0	2.09 ± 8.02	0.235 ^1^
Superior	86.77 ± 4.88	86.5 ± 4.02	−0.27 ± 2.57	0.623 ^1^
Superior nasal	88.64 ± 6.0	88.77 ± 4.58	0.14 ± 2.55	0.884 ^2^
Superior temporal	83.64 ± 5.62	83.59 ± 4.4	−0.05 ± 2.84	0.751 ^2^
Inferior	87.32 ± 5.78	86.05 ± 5.72	−1.27 ± 1.45	**0.001 ^1^**
Inferior nasal	88.5 ± 6.84	88.05 ± 6.28	−0.45 ± 2.3	0.14 ^2^
Inferior temporal	85.0 ± 6.59	84.36 ± 5.6	−0.64 ± 2.04	0.158 ^1^
**Disc analysis**				
QI	2.82 ± 0.94	4.39 ± 1.87	1.57 ± 1.85	**<0.001 ^2^**
Disc area (mm^2^)	2.36 ± 0.76	2.18 ± 0.55	−0.18 ± 0.64	0.25 ^2^
Vertical C/D	0.55 ± 0.24	0.49 ± 0.23	−0.06 ± 0.21	0.167 ^2^
RNFL thickness (µm)				
Average	93.15 ± 12.66	93.74 ± 9.51	0.59 ± 6.98	0.07 ^2^
Superior	106.5 ± 18.33	112.71 ± 16.07	6.21 ± 10.72	**0.005 ^1^**
Nasal	76.64 ± 14.6	74.5 ± 11.5	−2.14 ± 8.57	0.164 ^2^
Inferior	118.89 ± 21.87	120.82 ± 16.47	1.93 ± 14.62	0.085 ^2^
Temporal	68.75 ± 11.08	66.64 ± 9.95	−2.11 ± 6.31	**0.024 ^2^**

OCT: optical coherence tomography, SD: standard deviation, QI: quality index, GCIPL: ganglion cell-inner plexiform layer, C/D: cup to disc ratio, RNFL: retinal nerve fiber layer. *p* ^1^: paired *t*-test, *p* ^2^: Wilcoxon signed-rank test, *p* < 0.05 marked bold.

**Table 2:** Correlation analysis between age, topographic values, and OCT measurements without SCLs.

	Age (Years)		K1 (D)		K2 (D)		Kmax (D)		Astigmatism (D)	
	r	*p*	r	*p*	r	*p*	r	*p*	r	*p*
**Anterior segment analysis**										
QI	0.301	0.152	−0.393	**0.039**	−0.332	0.084	−0.471	**0.011**	−0.12	0.544
Corneal thickness (µm)										
Central	0.072	0.738	−0.09	0.648	−0.024	0.905	0.177	0.367	0.394	**0.038**
Maximum	0.387	0.062	0.179	0.361	0.189	0.336	0.395	**0.038**	0.068	0.73
Minimum	−0.189	0.375	0.028	0.887	0.102	0.606	0.288	0.137	0.393	**0.038**
Epithelial thickness (µm)										
Central	0.096	0.655	−0.501	**0.007**	−0.483	**0.009**	−0.42	**0.026**	0.043	0.828
Maximum	0.246	0.246	−0.318	0.099	−0.388	**0.041**	−0.268	0.168	−0.227	0.246
Minimum	−0.1	0.641	−0.273	0.159	−0.252	0.196	−0.198	0.311	0.152	0.44
**Ganglion analysis**										
QI	−0.39	0.08	−0.328	0.109	−0.46	**0.021**	−0.454	**0.023**	0.103	0.626
GCIPL thickness (µm)										
Average	−0.41	0.052	0.078	0.7	0.1	0.62	0.159	0.427	0.278	0.161
Minimum	−0.425	**0.043**	−0.323	0.101	−0.271	0.172	−0.253	0.203	0.179	0.371
Superior	−0.449	**0.031**	0.036	0.858	0.081	0.688	0.158	0.432	0.22	0.271
Superior nasal	−0.338	0.114	−0.007	0.972	−0.027	0.894	0.052	0.797	0.161	0.424
Superior temporal	−0.432	**0.039**	0.121	0.548	0.141	0.484	0.182	0.364	0.302	0.125
Inferior	−0.392	0.064	0.039	0.846	0.075	0.711	0.098	0.628	0.236	0.235
Inferior nasal	−0.334	0.119	−0.055	0.784	−0.043	0.832	0.009	0.965	0.192	0.338
Inferior temporal	−0.436	**0.037**	0.127	0.527	0.127	0.526	0.155	0.439	0.319	0.105
**Disc analysis**										
QI	−0.371	0.075	−0.348	0.07	−0.384	**0.044**	−0.403	**0.033**	0.163	0.407
Disc area (mm^2^)	0.128	0.551	−0.161	0.414	0.042	0.832	−0.032	0.873	0.463	**0.013**
Vertical C/D	0.188	0.389	0.061	0.763	0.135	0.502	0.162	0.421	0.155	0.439
RNFL thickness (µm)										
Average	−0.277	0.2	−0.552	**0.003**	−0.437	**0.023**	−0.498	**0.008**	0.173	0.388
Superior	−0.142	0.508	−0.213	0.276	−0.199	0.309	−0.266	0.172	−0.131	0.507
Nasal	−0.295	0.162	−0.509	**0.006**	−0.351	0.067	−0.386	**0.042**	0.444	**0.018**
Inferior	−0.039	0.855	−0.449	**0.017**	−0.429	**0.023**	−0.476	**0.01**	−0.162	0.411
Temporal	−0.494	**0.014**	0.056	0.777	0.101	0.611	0.133	0.501	0.376	**0.049**

OCT: optical coherence tomography, SCL: scleral contact lens, D: diopter, QI: quality index, GCIPL: ganglion cell-inner plexiform layer, C/D: cup to disc ratio, RNFL: retinal nerve fiber layer. Spearman’s correlation test, *p* < 0.05 marked bold.

**Table 3:** Correlation analysis between age, topographic values, and OCT measurements with SCLs.

	Age (Years)		K1 (D)		K2 (D)		Kmax (D)		Astigmatism (D)	
	r	*p*	r	*p*	r	*p*	r	*p*	r	*p*
**Anterior segment analysis**										
QI	0.344	0.163	−0.619	**0.003**	−0.605	**0.004**	−0.67	**0.001**	−0.409	0.066
Corneal thickness (µm)										
Central	0.359	0.143	0.055	0.814	0.154	0.505	0.276	0.227	0.343	0.129
Maximum	0.513	0.061	−0.042	0.874	0.171	0.512	0.366	0.149	0.47	0.057
Minimum	0.038	0.88	0.247	0.281	0.379	0.09	0.496	**0.022**	0.444	**0.044**
Epithelial thickness (µm)										
Central	0.741	**0.002**	−0.605	**0.01**	−0.616	**0.009**	−0.483	0.05	−0.177	0.496
Maximum	0.798	**0.001**	−0.481	0.05	−0.623	**0.008**	−0.4	0.112	−0.352	0.166
Minimum	−0.162	0.58	0.007	0.978	0.165	0.528	0.287	0.264	0.196	0.451
**Ganglion analysis**										
QI	−0.308	0.214	0.397	0.068	0.352	0.108	0.327	0.137	0.238	0.286
GCIPL thickness (µm)										
Average	−0.366	0.135	0.254	0.253	0.205	0.361	0.348	0.113	0.195	0.385
Minimum	−0.371	0.13	0.088	0.698	0.025	0.912	0.117	0.604	0.187	0.347
Superior	−0.233	0.352	0.274	0.217	0.24	0.283	0.348	0.113	0.138	0.54
Superior nasal	−0.289	0.246	0.216	0.334	0.146	0.516	0.255	0.252	0.049	0.827
Superior temporal	−0.211	0.401	0.234	0.295	0.148	0.51	0.236	0.29	0.109	0.628
Inferior	−0.359	0.143	0.19	0.396	0.203	0.364	0.33	0.134	0.267	0.23
Inferior nasal	−0.382	0.118	0.06	0.792	0.039	0.864	0.216	0.335	0.163	0.469
Inferior temporal	−0.348	0.157	0.291	0.189	0.171	0.447	0.329	0.135	0.151	0.502
**Disc analysis**										
QI	−0.513	**0.01**	0.013	0.946	0.058	0.769	0.05	0.8	0.414	**0.028**
Disc area (mm^2^)	0.047	0.829	−0.444	**0.018**	−0.298	0.124	−0.257	0.187	0.35	0.068
Vertical C/D	0.343	0.109	−0.095	0.638	−0.062	0.757	−0.053	0.794	−0.043	0.831
RNFL thickness (µm)										
Average	−0.307	0.145	−0.513	**0.005**	−0.402	**0.034**	−0.423	**0.025**	0.236	0.226
Superior	−0.45	**0.027**	−0.112	0.57	−0.037	0.851	−0.05	0.802	0.154	0.433
Nasal	−0.133	0.535	−0.578	**0.001**	−0.371	0.052	−0.425	**0.024**	0.504	**0.006**
Inferior	−0.146	0.495	−0.231	0.237	−0.217	0.267	−0.29	0.134	−0.059	0.765
Temporal	−0.263	0.214	0.13	0.51	0.111	0.574	0.166	0.397	0.221	0.259

OCT: optical coherence tomography, SCL: scleral contact lens, D: diopter, QI: quality index, GCIPL: ganglion cell-inner plexiform layer, C/D: cup to disc ratio, RNFL: retinal nerve fiber layer. Spearman’s correlation test, *p* < 0.05 marked bold.

## Data Availability

The data presented in this study are available on request from the corresponding author due to ethical reasons.
